# Sociodemographic and clinical characteristics of individuals exposed to smoking or biomass smoke and followed at primary health care centers in Brazil: a multicenter study

**DOI:** 10.36416/1806-3756/e20250001

**Published:** 2025-06-20

**Authors:** Juliana O Barros, Flavio F Arbex, Alcindo Cerci, Leandro G Fritscher, Suzana E Tanni, Gerson F Souza, Oliver A Nascimento, José R Jardim

**Affiliations:** 1. Universidade Federal de São Paulo, Escola Paulista de Medicina, São Paulo (SP) Brasil.; 2. Universidade de Araraquara - UNIARA - Araraquara (SP) Brasil.; 3. Universidade Estadual de Londrina, Londrina (PR) Brasil.; 4. Pontifícia Universidade Católica de Porto Alegre, Porto Alegre (RS) Brasil.; 5. Universidade Estadual de São Paulo, Botucatu (SP) Brasil.; 6. Universidade Federal do Rio Grande do Norte, Natal (RN) Brasil.; 7. Faculdade de Medicina São Leopoldo Mandic, Campinas (SP), Brasil.

**Keywords:** Smoking, Risk factors, Biomass, Primary health care

## Abstract

**Objective:**

To describe the sociodemographic and clinical characteristics of individuals exposed to smoking or biomass smoke and followed at primary health care (PHC) centers across three states in Brazil.

**Methods:**

This was a cross-sectional multicenter study including patients followed at any of four PHC centers in Brazil. Patients ≥ 35 years of age who were smokers or former smokers, or were exposed to biomass smoke were included, the exception being those with physical/mental disabilities and those who were pregnant. Face-to-face assessments included a questionnaire assessing clinical and sociodemographic characteristics, as well as the COPD Assessment Test (CAT) and the modified Medical Research Council (mMRC) dyspnea scale.

**Results:**

Of a total of 737 patients, 56.3% were female and 64.2% were White, with a mean age of 57.7 ± 11.8 years. Most (54.4%) had < 9 years of schooling, 50.2% had low socioeconomic status, and 71.5% were overweight/obese. Smokers accounted for 43.4% of the study sample, whereas 15.0% had no direct exposure to cigarette smoke. Common symptoms included cough, in 37.3%; wheezing, in 33.8%; and phlegm, in 27.4%. Most (75.1%) of the study participants had mMRC dyspnea scale scores of 0 or 1. CAT scores were 0-10, in 40.2%; 11-20, in 44.6%; 21-30, in 14.1%; and 31-40, in 1.1%. Binary logistic regression showed that sex and age significantly impacted mMRC dyspnea scale predictions, whereas BMI and socioeconomic status influenced CAT predictions. Common comorbidities included hypertension, in 51.3%; depression, in 27.4%; and diabetes, in 24.3%. No association was found between hypertension and obesity or smoking, or between diabetes and obesity or smoking.

**Conclusions:**

PHC patients with risk factors such as smoking and exposure to biomass smoke have a high comorbidity burden, with over half experiencing mild to moderate quality-of-life impacts. This study emphasizes the need for targeted preventive measures in PHC settings.

## INTRODUCTION

Health care in the Brazilian Unified Health Care System is organized into three levels: primary care, secondary care, and tertiary care.^1^ Primary health care (PHC) relies on low-density technology and is provided through PHC centers and family health care centers. 

PHC is the main entry point to the Brazilian Health Care Network. Most PHC centers address chronic conditions, with 21 of the 28 most common conditions (82%) being chronic. Only 5.7% of all PHC visits focus on prevention and health maintenance, highlighting a predominant emphasis on acute conditions or exacerbated chronic diseases.^2^

Cardiovascular diseases, arterial conditions, certain cancers, and respiratory diseases such as COPD share common risk factors, mainly smoking and exposure to air pollutants such as biomass smoke and occupational dust.^3^ ^-^ ^4^ Many result from long-term smoking, and, if organ damage occurs, disease progression may continue despite smoking cessation. This is particularly true for COPD, in which inflammation remains progressive once initiated.^5^

Assessing the profile of PHC patients is crucial for early interventions to prevent or slow disease progression, alleviate symptoms, and improve quality of life.^6^ Identifying patients with risk factors such as smoking and biomass exposure enables early diagnosis, benefiting the health care system and patients. The objective of the present study was to describe the sociodemographic and clinical characteristics of individuals exposed to smoking or biomass smoke and followed at PHC centers across three states in Brazil. 

## METHODS

The present study was approved by the Research Ethics Committee of the *Universidade Federal de São Paulo* (CAAE 81033317.8.1001.5505; Ruling no. 3,618,999) and was registered at ClinicalTrials.gov under NCT03018808. 

The study was conducted at four PHC centers without specialists in respiratory medicine. Patients ≥ 35 years of age attending PHC centers for spontaneous or scheduled routine visits in the cities of Porto Alegre, Londrina, Araraquara, and Botucatu, Brazil, were invited to participate in the study. 

### Inclusion criteria

The inclusion criteria were as follows: being ≥ 35 years of age; being a current or former smoker (having smoked ≥ 100 cigarettes in their lifetime); and having been exposed to biomass smoke (having been exposed to biomass smoke for ≥ 100 h in their lifetime). 

### Exclusion criteria

The exclusion criteria were as follows: having mental or physical impairments; having a heart rate ≥ 120 bpm; currently receiving treatment for tuberculosis; concurrently participating in a clinical trial; being pregnant; and having any contraindication to spirometry. 

### Assessment

Eligible patients who gave written informed consent completed a standardized questionnaire adapted from the *Proyecto Latinoamericano de Investigación en Obstrucción Pulmonar* (PLATINO, Latin American Project for the Investigation of Obstructive Lung Disease) study,^7^ the COPD Assessment Test (CAT),^8^ and the modified Medical Research Council (mMRC) dyspnea scale.^9^ Briefly, the data collection included the following: sociodemographic characteristics; respiratory symptoms in the past 12 months; a history of atopy; self-reported disease diagnosis; smoking status; environmental exposures; socioeconomic status, in accordance with the Brazilian Institute of Geography and Statistics criteria^10^; and BMI, calculated by collecting data on patient weight and height. A patient was considered to have a prior diagnosis of COPD if they reported having been diagnosed with chronic bronchitis, emphysema and/or COPD by a physician and if they were ≥ 35 years of age at the time of diagnosis. 

### Statistical analysis

Descriptive and inferential analyses were conducted with the IBM SPSS Statistics software package, version 22 (IBM Corp., Armonk, NY, USA). The Shapiro-Wilk test evaluated the distribution of the data. Numerical variables were described as means and standard deviations, and categorical variables were expressed as absolute numbers and proportions. All statistical inference tests were performed with bootstrap sampling (1,000 replicates), allowing the use of parametric tests. This is a robust and reliable method to provide valid confidence intervals when normal distribution of residuals is not observed and/or the sample is small.^11^ The level of significance was set at p < 0.05 (two-tailed) for all tests. 

Numerical variables were compared by means of one-way ANOVA (for three or more groups) and the unpaired t-test (for two groups), and categorical variables were analyzed by means of Pearson’s chi-square test or Fisher’s exact test, as appropriate. To minimize the risk of type II errors, effect sizes were calculated using Hedges’ *g* for comparisons made with ANOVA and the unpaired t-test. The effect size was interpreted as weak (< 0.39), moderate (0.40-0.69), strong (0.70-0.99), or perfect (= 1). For comparisons made with Pearson’s chi-square test, the effect size was calculated by Cramér’s V, being interpreted as weak (> 0.05), moderate (> 0.10), strong (> 0.15), or very strong (> 0.25).^12^

A binary logistic regression analysis (enter method) was performed to investigate the extent to which the occurrence of dyspnea, as assessed by the mMRC dyspnea scale, and the impact of respiratory symptoms on daily life, as assessed by the CAT, could be predicted by sociodemographic characteristics (including sex, age, BMI, the Charlson Comorbidity Index, socioeconomic status [class A, B, C, or D/E], and smoking history). 

## RESULTS

A total of 737 patients were included in the present study. Of those, 56.3% were female, and approximately two thirds were White. The mean age of the study participants was 57.7 ± 11.8 years. Most had < 9 years of schooling; belonged to socioeconomic class C1, C2, or D/E; and were predominantly overweight or obese (Table 1). 

**Table 1:** Sociodemographic characteristics of individuals exposed to smoking or biomass smoked and followed at any of four primary health care centers in Brazil.^a^

Variable	N = 737
Sex	
Male	322 (43.7)
Female	415 (56.3)
Skin color	
White	473 (64.2)
Non-White	264 (35.8)
Age, years	57.7 ± 11.8
Weight, kg	76.1 ± 16.0
Height, m	1.64 ± 0.1
BMI, kg/m^2^	28.2 ± 5.5
Nutritional status	
Underweight	10 (1.4)
Normal	200 (27.1)
Overweight	282 (38.3)
Obese	245 (33.2)
Level of education	
Illiterate/< 9 years of schooling	197 (26.7)
= 9 years of schooling/incomplete high school education	361 (49.0)
Complete high school education	149 (20.2)
Higher education	30 (4.1)
Socioeconomic status^b^	
Class A	9 (1.2)
Class B1/B2	97 (13.2)
Class C1/C2	468 (63.5)
Class D/E	163 (22.1)
People living in the same household	
1	104 (14.1)
2	240 (32.6)
3	196 (26.6)
≥ 4	196 (26.6)

a Data are presented as n (%) or mean ± SD. ^b^In accordance with the Brazilian Institute of Geography and Statistics criteria,^10^ as follows: socioeconomic class A, 45-100 points; socioeconomic class B1, 38-44 points; socioeconomic class B2, 29-37 points; socioeconomic class C1, 23-28 points; socioeconomic class C2, 17-22 points; and socioeconomic class D/E, 0-16 points.

According to the mMRC dyspnea scale, 33.4% of the study participants had dyspnea on exertion only (a score of 0); whereas 41.7%, 13.7%, 8.3%, and 3.0%, respectively, had a score of 1, 2, 3, and 4. With regard to the impact of respiratory symptoms on patient health status, as assessed by the CAT, 40.2% experienced no impact (a score of 0-10), whereas 44.6%, 14.1%, and 1.1%, respectively, experienced moderate (a score of 11-20), severe (a score of 21-30), and very severe (a score of 31-40) impacts. 

The binary logistic regression model for predicting the occurrence of dyspnea was statistically significant (χ²(8) = 28.570; p < 0.001; Nagelkerke’s *R²* = 0.067), correctly predicting 73.2% of cases (98.9% of cases correctly classified for those with an mMRC dyspnea scale score of 0 or 1 and 1.9% for those with an mMRC dyspnea scale score ≥ 2). Of all predictors analyzed, only sex and age had a significant impact on mMRC dyspnea scale predictions. Men were 1.94 times more likely to have an mMRC dyspnea scale score ≥ 2 than were women, and for each additional year of age, the likelihood of an mMRC dyspnea scale score ≥ 2 increased by 1.03 (Table 2). 

**Table 2:** Influence of sociodemographic variables in the logistic regression model for predicting the occurrence of dyspnea, as assessed by the modified Medical Research Council dyspnea scale, in individuals exposed to smoking or biomass smoke and followed at any of four primary health care centers in Brazil.

	Wald	df	p	Exp(B)	95% CI for Exp(B)	
					Lower	Upper
Sex, male	10.469	1	< 0.001	1.935	0.245	1.140
Age, years	10.903	1	0.003	1.028	0.011	0.048
BMI, kg/m^2^	4.030	1	0.057	1.034	0.002	0.073
Pack-years	0.414	1	0.492	0.998	−0.008	0.004
Charlson Comorbidity Index	1.252	1	0.263	0.284	−0.143	0.037
Socioeconomic status						
Class A	0.514	1	0.473	0.236	−20.437	0.874
Class B	0.203	1	0.652	0.686	−0.850	0.492
Class C	1.868	1	0.172	0.184	−0.716	0.113
Constant	22.089	1	< 0.001	0.027	−5.387	−2.149

df: degrees of freedom.

Regarding the CAT, the model was also statistically significant (χ²(8) = 46.619; p < 0.001; Nagelkerke’s *R²* = 0.102), with the predictors correctly classifying 68.9% of cases (23.9% of cases correctly classified for those with a CAT score of < 10 and 93.4% for those with a CAT score ≥ 10). Only BMI and socioeconomic status had a significant influence on predicting the impact of respiratory symptoms on health status. For each one-unit increase in the BMI, the likelihood of impact on health status increased by 1.05 times. Regarding socioeconomic status, patients belonging to socioeconomic class A were 0.20 times less likely to experience an impact on health status than were those belonging to socioeconomic class D/E; patients belonging to socioeconomic class B were 0.18 times less likely to experience an impact on health status than were those belonging to socioeconomic class D/E; and patients belonging to socioeconomic class C were 0.61 times less likely to experience an impact on health status than were those belonging to socioeconomic class D/E (Table 3). 

**Table 3:** Influence of sociodemographic variables in the logistic regression model for predicting the impact of respiratory symptoms on activities of daily living, as assessed by the COPD Assessment Test, in individuals exposed to smoking or biomass smoke and followed at any of four primary health care centers in Brazil.

	Wald	df	p	Exp(B)	95% CI for Exp(B)	
					Lower	Upper
Sex, male	3.247	1	0.070	1.387	−0.044	0.706
Age, years	0.003	1	0.943	1.000	−0.015	0.017
BMI, kg/m^2^	6.574	1	0.009	1.045	0.009	0.082
Pack-years	0.026	1	0.906	1.000	−0.004	0.009
Charlson Comorbidity Index	0.236	1	0.622	0.981	−0.102	0.073
Socioeconomic status						
Class A	4.328	1	0.017	0.201	−21.942	−0.061
Class B	28.633	1	< 0.001	0.179	−2.397	−1.126
Class C	4.541	1	0.032	0.610	−0.973	−0.043
Constant	0.117	1	0.732	0.784	−1.643	1.032

df: degrees of freedom.

The diseases that were most commonly reported by the study participants were hypertension, in 51.3%; depression, in 27.4%; diabetes mellitus, in 24.3%; rhinitis, in 20.8%; asthma, in 16.8%; COPD, in 6.8%; and tuberculosis, in 3.8%. There was no association between having a diagnosis of hypertension and obesity (χ²(1) = 0.499; p = 0.480; Cramér’s V = 0.026) or smoking status (χ²(2) = 0.845; p = 0.655; Cramér’s V = 0.034). Similarly, no association was found between having a diagnosis of diabetes mellitus and obesity (χ²(1) = 0.266; p = 0.606; Cramér’s V = 0.019) or smoking status (χ²(2) = 0.085; p = 0.958; Cramér’s V = 0.011). The comparison between patients with and without a prior diagnosis of COPD showed that the proportion of men was significantly higher than that of women in the COPD group. Additionally, patients with COPD had a significantly higher smoking history. No significant differences were observed for the remaining variables (Table 4). 

**Table 4:** Comparison of sociodemographic and clinical variables between individuals with and without a prior diagnosis of COPD followed at any of four primary health care centers in Brazil.^a^

	Prior diagnosis of COPD*		p	Effect Size
	No (n = 653)	Yes (n = 50)		
Sex				
Male	280 (42.9)	31 (62.0)	0.011	0.10
Female	373 (57.1)	19 (38.0)		
Age, years	57.9 ± 11.7	57.7 ± 12.7	0.926	0.01
BMI, kg/m^2^	28.2 ± 5.5	29.2 ± 6.0	0.248	0.19
Pack-years	35.2 ± 32.7	47.1 ± 34.3	0.039	0.36
mMRC dyspnea scale score	1.1 ± 1.0	1.0 ± 0.9	0.528	0.09
CAT score	12.9 ± 7.7	12.3 ± 6.7	0.521	0.09
Socioeconomic status				
Class A	9 (1.4%)	0 (0.0%)	0.896	0.03
Class B	87 (13.3%)	7 (14.0%)		
Class C	414 (63.4%)	31 (62.0%)		
Class D/E	143 (21.9%)	12 (24.0%)		

mMRC: modified Medical Research Council; and CAT: COPD Assessment Test. ^a^Data are presented as n (%) or mean ± SD. *Of the 737 individuals in the sample, 34 responded “I don’t know” or “I don’t remember” to the question of whether a physician had ever diagnosed them with COPD or chronic bronchitis and were therefore excluded from the analysis.

Patients reported experiencing the following respiratory symptoms over the past 12 months: cough, in 37.3%; phlegm, in 27.4%; dyspnea, in 25.0%; wheezing, in 33.8%; and both wheezing and dyspnea, in 15.1%. Table 5 describes smoking status and exposure to biomass smoke. Approximately 15% of the patients had no direct contact with cigarette smoke, whereas 42% were exposed to secondhand smoke and 10% were exposed to biomass smoke. A high prevalence of smoking history was observed, both in terms of pack-years and the number of years smoked. The comparison between patients classified as exposed to biomass smoke and those classified as not exposed to biomass smoke showed that the former had significantly higher CAT scores than did the latter. No significant differences were observed for the other variables (Table 6). 

**Table 5:** Environmental exposure and smoking status in individuals followed at any of four primary health care centers in Brazil.^a^

Variable	N = 737
Smoking status	
Former smoker	306 (41.5)
Current smoker	320 (43.4)
Never smoker	111 (15.1)
Smoking history, pack-years	35.7 ± 32.7
Smoking duration, years	31.8 ± 14.1
Received smoking cessation advice	287 (38.9)
Secondhand smoke exposure	
Yes	313 (42.5)
No	424 (57.5)
Exposure to biomass smoke	
Yes	74 (10.0)
No	654 (88.6)
Don’t know/Don’t remember	9 (1.2)

a Data are presented as n (%) or mean ± SD.

**Table 6:** Comparison of sociodemographic and clinical variables between individuals with and without environmental exposure to biomass smoke and followed at any of four primary health care centers in Brazil.^a^

	Exposure to biomass smoke*		p	Effect size
	No (n = 654)	Yes (n = 74)		
Sex				
Male	287 (43.9)	32 (43.2)	> 0.999	0.004
Female	367 (56.1)	42 (56.8)		
Age, years	57.7 ± 11.7	58.1 ± 12.3	0.798	0.03
BMI, kg/m^2^	28.1 ± 5.6	29.1 ± 5.3	0.131	0.18
Pack-years	34.9 ± 28.3	44.9 ± 59.7	0.260	0.31
mMRC dyspnea scale score	1.0 ± 1.0	1.2 ± 1.2	0.102	0.20
CAT score	12.7 ± 7.4	15.0 ± 8.4	0.032	0.29
Socioeconomic status				
Class A	8 (1.2)	1 (1.4)	0.556	0.05
Class B	85 (13.0)	11 (14.9)		
Class C	412 (63.0)	50 (67.6)		
Class D/E	149 (22.8)	12 (16.2)		

mMRC: modified Medical Research Council; and CAT: COPD Assessment Test. ^a^Data are presented as n (%) or mean ± SD. *Of the 737 individuals in the sample, 9 responded “I don’t know” or “I don’t remember” to the question of whether they had been exposed to biomass smoke and were therefore excluded from the analysis.

A comparison of clinical characteristics among patients revealed that former smokers were significantly younger and had significantly lower BMI than never smokers and current smokers. CAT scores were significantly lower in never smokers than in former smokers. No differences were observed among the groups regarding mMRC dyspnea scale scores (supplementary material, Table S1). 

## DISCUSSION

Most of the patients exposed to smoking or biomass smoke and followed at PHC centers across four cities in Brazil were female; were White; had low socioeconomic status; were overweight or obese; and had a low level of education. 

We found that 56% of the patients participating in the present study were female. Data from 81 randomized PHC clinical trials from the Netherlands, the USA, the UK, and Spain also revealed a predominance of women, with rates between 55% and 60%.^13^ Studies conducted in the city of Goiânia, in central-western Brazil, reported a prevalence of women ranging from 39% to 71%.^14^ The frequency of medical visits has been reported to be 1.90 to 2.43 times higher in women than in men.^15^ Reasons for this disparity are not clear but may include the perception that men seeking health care are demonstrating weakness, fear, or insecurity, which contrasts with the idealized notion of male invulnerability. Additionally, work schedules often conflict with health care service hours, posing a barrier for men.^16^ The fact that women tend to assess their own health status as being worse might explain their higher demand for health services. In addition, a double burden of work and household chores, particularly among low-income women, combined with psychological and emotional exhaustion, likely further increase their health care needs.^17^

Our sample predominantly came from peripheral PHC centers, with 85.6% of patients being classified as belonging to low socioeconomic classes and one quarter being illiterate or having had < 9 years of schooling, findings that are in accordance with those of the 2013 and 2019 Brazilian National Health Surveys. This vulnerable population remains heavily reliant on PHC.^18^ Brazil displays significant disparities in social class, level of education, and access to health care, and PHC plays an essential role in promoting equity among those populations. There is a direct relationship between education levels and health care utilization. The PLATINO study found that 54.3% of patients in the city of São Paulo, Brazil, had had ≤ 4 years of schooling,^19^ and the 2019 Brazilian National Household Sample Survey found that 51.2% of the Brazilian population > 25 years of age had had < 9 years of schooling.^18^

In a study published in 2023,^19^ low-income populations in 20 cities in Brazil were reported to have better access to PHC facilities than did high-income populations accessing private services; this is largely due to a wide distribution of PHC centers and prioritization of underserved areas in the Brazilian national PHC network. However, Black populations still face more barriers in accessing PHC than do White populations.^20^

In our study, 71.5% of the patients were classified as being overweight or obese on the basis of their BMI, a finding that underscores an urgent need for care strategies for patients with excess weight. In a meta-analysis published in 2022, excess weight in adults in Brazil was reported to have increased from 33.5% in the 1974-1990 period (95% CI, 25.0-42.6) to 52.5% in the 2011-2020 period (95% CI, 47.6-57.3).^21^ However, according to the Health Information System for PHC in Brazil, obesity accounts for < 3% of all conditions evaluated in over 105 million consultations.^22^ The 2019 Telephone-based System for the Surveillance of Risk and Protective Factors for Chronic Diseases showed a high prevalence of overweight and obesity in Brazilian state capitals, ranging from 49.1% in the city of Vitória to 60.9% in the city of Manaus.^23^ Obesity has been reported to be most prevalent in non-White women with low education levels and men in the 40- to 59-year age bracket with average incomes.^24^ Similar trends have been reported in PHC settings in the city of São José dos Pinhais, Brazil, where 67.3% have been classified as being overweight or obese.^25^ Overweight and obese patients tend to utilize health care services more frequently because of a higher prevalence of comorbidities such as hypertension and diabetes mellitus.^26^ In the PLATINO study, overweight and obesity rates ranged from 54.5% in São Paulo, Brazil, to 68.5% in Santiago, Chile.^19^

Although our sample had a high prevalence of current smokers (43.4%), it is important to note that smoking was a criterion for inclusion in the study. Smoking cessation programs are highly cost-effective, extending survival by 10-15 years and reducing the risk of chronic noncommunicable diseases associated with tobacco.^27^ Even brief advice to quit smoking, without pharmacological intervention, improves cessation rates and positively impacts lung function and quality of life.^28^ Although official smoking cessation programs provide guidance and medications at no cost in Brazil, fewer than 5% of smokers receive this treatment.^29^ In our sample, 42.5% of the patients were found to be exposed to secondhand smoke. According to the WHO, 33% of men, 35% of women, and 40% of children worldwide are exposed to secondhand smoke.^30^ Data from the 2013 and 2019 Brazilian National Health Surveys show a reduction in secondhand smoke exposure at home (by 1.6%) and in the workplace (by 5%), with higher exposure among patients with lower education levels.^31^

We found that 75% of the patients in the present study had an mMRC dyspnea scale score of 0-1, and 80% had a CAT score of 0-20, indicating low to moderate levels of dyspnea and disease impact. Although several studies have assessed COPD symptoms in PHC settings, few have evaluated dyspnea using the mMRC dyspnea scale or disease impact using the CAT. Studies conducted in the UK, Greece, and Spain reported similar findings, with approximately 50% of patients showing mild dyspnea (a median MRC scale score of ≤ 2).^32^ ^-^ ^34^ Although our logistic regression model including sociodemographic variables showed high accuracy in classifying patients without dyspnea (98.9%), its performance in correctly identifying those with dyspnea was extremely low (1.9%). This suggests that variables other than sociodemographic variables play a more relevant role in predicting dyspnea among patients with risk factors such as smoking and exposure to biomass smoke in PHC settings and should be considered in future analyses. In contrast, our model for predicting CAT scores performed better in terms of correctly identifying patients in whom respiratory symptoms had an impact on activities of daily living (93.4%), with only BMI and socioeconomic status having a significant influence. It is expected that patients from higher socioeconomic classes have greater access to health care; on the other hand, the impact of increased BMI on activities of daily living may be due to excess weight itself and obesity-related diseases. 

One third of our sample reported at least one respiratory symptom, with cough being the most common (37.3%), followed by wheezing (33.8%). Respiratory symptoms are common in smokers because of airway inflammation and persistent airflow limitation.^35^ In the city of São Paulo, the PLATINO study found that 54.8% of individuals reported at least one respiratory symptom, with wheezing and dyspnea being the most common (34.9%).^7^ In a study conducted in central-western Brazil, symptom burden was found to be high in patients with risk factors for COPD, with symptoms including dyspnea (58.4%), sputum production (39.4%), and chronic cough (35%).^36^ Current smokers were significantly more likely to experience severe dyspnea, productive cough, and exertional dyspnea than were former smokers or never smokers.^36^ Patients exposed to biomass smoke had higher CAT scores than did those without exposure to biomass smoke, indicating a greater impact on their quality of life.^36^ Similarly, Mexican women exposed to biomass smoke have been reported to have more respiratory symptoms,^37^ a finding corroborated by a study conducted in China and reporting that COPD patients exposed to biomass smoke alone had higher CAT scores than did those exposed to tobacco alone or occupational hazards alone (17.5 ± 6.3 vs. 15.3 ± 6.3 vs. 15.2 ± 6.3; p < 0.05).^38^

In addition to respiratory symptoms, comorbidities were common in our sample. Hypertension affected one in two patients, whereas one in four reported depression or diabetes mellitus. Shared risk factors such as physical inactivity and smoking, as well as systemic inflammation and oxidative stress, contribute to these comorbidities. Although our sample did not show an association of hypertension and diabetes mellitus with smoking and obesity, it is well known that systemic inflammation contributes to the development of insulin resistance and the onset of diabetes mellitus.^39^ Smokers are twice as likely to develop diabetes mellitus as are nonsmokers, the risk increasing with greater smoking intensity.^40^ The acute effects of smoking on blood pressure include transient sympathetic activation; however, the chronic mechanisms remain unclear. 

The present study was conducted in four PHC centers across different regions of Brazil, specifically targeting patients with risk factors such as smoking and exposure to biomass smoke, showing a high prevalence of respiratory symptoms, hypertension, and diabetes mellitus. These findings underscore the importance of evaluating risk factors for prevalent diseases so as to allow early diagnosis and intervention. 

The present study has some limitations. Although it was originally designed to include data from other regions of Brazil, this was not possible, because of the COVID-19 pandemic. Although several PHC centers in other states were contacted, they could not initiate data collection. Because the study was conducted in four PHC centers located either in southeastern Brazil or in southern Brazil, it could not fully capture the diversity of PHC users across the country. The high smoking prevalence observed in the present study cannot be considered representative of the regions, because smoking was one of the inclusion criteria. The fact that there was a large number of patients with lower socioeconomic status and lower education levels may limit the generalizability of the findings to other populations. Finally, the information collected was self-reported through questionnaires. Although this is needed in order to investigate the frequency and severity of symptoms, it may be subject to biases. 

The evaluation of patients with risk factors such as smoking and exposure to biomass smoke in PHC settings revealed a predominance of women with low socioeconomic status and low education levels, as well as a high prevalence of respiratory symptoms, hypertension, and diabetes mellitus. These findings are crucial for understanding and developing public health policies focusing on risk factors, allowing early diagnosis and timely interventions. 

## References

[1] Gonçalves MA (2021). Organização e funcionamento do SUS.

[2] Brasil (2015). Conselho Nacional de Secretários de Saúde. A Atenção Primária e as Redes de Atenção à Saúde.

[3] Wilcox NS, Amit U, Reibel JB, Berlin E, Howell K, Ky B (2024). Cardiovascular disease and cancer shared risk factors and mechanisms. Nat Rev Cardiol.

[4] Parris BA, O'Farrell HE, Fong KM, Yang IA (2019). Chronic obstructive pulmonary disease (COPD) and lung cancer common pathways for pathogenesis. J Thorac Dis.

[5] Adcock IM, Caramori G, Barnes PJ (2011). Chronic obstructive pulmonary disease and lung cancer new molecular insights. Respiration.

[6] Global Initiative for Chronic Obstructive Lung Disease [homepage on the Internet] (c2024). Global strategy for the diagnosis, management, and prevention of chronic obstructive pulmonary disease: GOLD Executive Summary 2024.

[7] Nascimento OA, Camelier A, Rosa FW, Menezes AM, Pérez-Padilla R, Jardim JR (2007). Chronic obstructive pulmonary disease is underdiagnosed and undertreated in São Paulo (Brazil) results of the PLATINO study. Braz J Med Biol Res.

[8] Silva GP, Morano MT, Viana CM, Magalhães CB, Pereira ED (2013). Portuguese-language version of the COPD Assessment Test validation for use in Brazil. J Bras Pneumol.

[9] Kovelis D, Segretti NO, Probst VS, Lareau SC, Brunetto AF, Pitta F (2008). Validation of the modified pulmonary functional status and dyspnea questionnaire and the Medical Research Council scale for use in Brazilian patients with chronic obstructive pulmonary disease. J Bras Pneumol.

[10] Brasil. Instituto Brasileiro de Geografia e Estatística (IBGE) [homepage on the Internet] (c2022). Síntese de indicadores sociais: uma análise das condições de vida da população brasileira: 2022.

[11] LaFleur BJ, Greevy RA (2009). Introduction to permutation and resampling-based hypothesis tests. J Clin Child Adolesc Psychol.

[12] Akoglu H (2018). User's guide to correlation coefficients. Turk J Emerg Med.

[13] Lindson N, Pritchard G, Hong B, Fanshawe TR, Pipe A, Papadakis S (2021). Strategies to improve smoking cessation rates in primary care. Cochrane Database Syst Rev.

[14] Pereira AS (2013). Prevalência de Doença Pulmonar Obstrutiva Crônica e de seu subdiagnóstico em pacientes hipertensos do Programa HIPERDIA de uma unidade de atenção primária à saúde na cidade de Goiânia.

[15] Levorato CD, Mello LM, Silva AS, Nunes AA (2014). Factors associated with the demand for health services from a gender-relational perspective [Article in Portuguese. Cien Saude Colet.

[16] Gomes R, Nascimento EF, Araújo FC (2007). Why do men use health services less than women Explanations by men with low versus higher education. Cad Saude Publica.

[17] Cobo B, Cruz C, Dick PC (2021). Gender and racial inequalities in the access to and the use of Brazilian health services. Cien Saude Colet.

[18] Giovanella L, Bousquat A, Schenkman S, Almeida PF, Sardinha LMV, Vieira MLFP (2021). The Family Health Strategy coverage in Brazil what reveal the 2013 and 2019 National Health Surveys Cien Saude. Colet.

[19] Tomasiello D, Bazzo JP, Parga JP, Servo L, Pereira RHM (c2023). Desigualdades raciais e de renda no acesso à saúde nas cidades brasileiras [monograph on the Internet].

[20] Kodaira K, Abe FC, Galvão TF, Silva MT (2021). Time-trend in excess weight in Brazilian adults: A systematic review and meta-analysis. PLoS One.

[21] Brasil (2022). Ministério da Saúde. Secretaria de Atenção Primária à Saúde. Departamento de Promoção à Saúde. Manual de atenção às pessoas com sobrepeso e obesidade no âmbito da Atenção Primária à Saúde (APS) do Sistema Único de Saúde.

[22] Brasil. Ministério da Saúde (c2020). Vigitel Brasil 2019: vigilância de fatores de risco e proteção para doenças crônicas por inquérito telefônico: estimativas sobre frequência e distribuição sociodemográfica de fatores de risco e proteção para doenças crônicas nas capitais dos 26 estados brasileiros e no Distrito Federal em 2019 [monograph on the Internet].

[23] Watanabe LM, Delfino HBP, Pinhel MAS, Noronha NY, Diani LM, Assumpção LCP (2022). Food and Nutrition Public Policies in Brazil From Malnutrition to Obesity. Nutrients.

[24] Santos LPD, Silva ATD, Rech CR, Fermino RC (2021). Physical Activity Counseling among Adults in Primary Health Care Centers in Brazil. Int J Environ Res Public Health.

[25] Rimes-Dias KA, Costa JC, Canella DS (2022). Obesity and health service utilization in Brazil data from the National Health Survey. BMC Public Health.

[26] Menezes AM, Perez-Padilla R, Jardim JR, Muiño A, Lopez MV, Valdivia G (2005). Chronic obstructive pulmonary disease in five Latin American cities (the PLATINO study) a prevalence study. Lancet.

[27] Ministério da Saúde. Instituto Nacional de Câncer José Alencar Gomes Silva (c2019). Coordenação de Prevenção e Vigilância. Benefícios obtidos após parar de fumar: manual do participante - Sessão 4 [monograph on the Internet].

[28] Han M, Fu Y, Ji Q, Deng X, Fang X (2023). The effectiveness of theory-based smoking cessation interventions in patients with chronic obstructive pulmonary disease a meta-analysis. BMC Public Health.

[29] Silva LC, Araújo AJ, Queiroz ÂM, Sales MD, Castellano MV, Comissão de Tabagismo da SBPT (2016). Smoking control challenges and achievements. J Bras Pneumol.

[30] Oberg M, Jaakkola MS, Woodward A, Peruga A, Prüss-Ustün A (2011). Worldwide burden of disease from exposure to second-hand smoke a retrospective analysis of data from 192 countries. Lancet.

[31] Malta DC, Gomes CS, Andrade FMD, Prates EJS, Alves FTA, Oliveira PPV (2021). Tobacco use, cessation, secondhand smoke and exposure to media about tobacco in Brazil results of the National Health Survey 2013 and 2019. Rev Bras Epidemiol.

[32] Josephs L, Culliford D, Johnson M, Thomas M (2019). COPD overdiagnosis in primary care a UK observational study of consistency of airflow obstruction. NPJ Prim Care Respir Med.

[33] Llordés M, Jaén A, Almagro P, Heredia JL, Morera J, Soriano JB (2015). Prevalence, Risk Factors and Diagnostic Accuracy of COPD Among Smokers in Primary Care. COPD.

[34] Stafyla E, Kotsiou OS, Deskata K, Gourgoulianis KI (2018). Missed diagnosis and overtreatment of COPD among smoking primary care population in Central Greece old problems persist. Int J Chron Obstruct Pulmon Dis.

[35] Celli B, Fabbri L, Criner G, Martinez FJ, Mannino D, Vogelmeier C (2022). Definition and Nomenclature of Chronic Obstructive Pulmonary Disease: Time for Its Revision. Am J Respir Crit Care Med.

[36] Queiroz MC, Moreira MA, Rabahi MF (2012). Underdiagnosis of COPD at primary health care clinics in the city of Aparecida de Goiânia, Brazil. J Bras Pneumol.

[37] Ramírez-Venegas A, Velázquez-Uncal M, Pérez-Hernández R, Guzmán-Bouilloud NE, Falfán-Valencia R, Mayar-Maya ME (2018). Prevalence of COPD and respiratory symptoms associated with biomass smoke exposure in a suburban area. Int J Chron Obstruct Pulmon Dis.

[38] Duan JX, Cheng W, Zeng YQ, Chen Y, Cai S, Li X (2020). Characteristics of Patients with Chronic Obstructive Pulmonary Disease Exposed to Different Environmental Risk Factors A Large Cross-Sectional Study. Int J Chron Obstruct Pulmon Dis.

[39] Kondo T, Nakano Y, Adachi S, Murohara T (2019). Effects of Tobacco Smoking on Cardiovascular Disease. Circ J.

[40] Feary JR, Rodrigues LC, Smith CJ, Hubbard RB, Gibson JE (2010). Prevalence of major comorbidities in subjects with COPD and incidence of myocardial infarction and stroke a comprehensive analysis using data from primary care. Thorax.

